# Clinical values of urinary IL-6 in asymptomatic renal hematuria and renal hematuria with proteins

**DOI:** 10.3892/etm.2013.1124

**Published:** 2013-05-20

**Authors:** MINGHUI SONG, LU MA, DAN YANG, ZHIJUN HE, CHAOBO LI, TAO PAN, ANJUN LI

**Affiliations:** Integrated Chinese and Western Medicine Treatment of Renal Disease Centre, Beidaihe Sanatorium of Beijing Military Area Command, Qinhuangdao, Hebei 066100, P.R. China

**Keywords:** interleukin-6, renal hematuria, 24 h urinary protein

## Abstract

Renal hematuria is caused by glomerular disease. Under pathological conditions, the distribution of interleukin-6 (IL-6) in kidney tissue is abnormal and urinary IL-6 levels are increased. Abnormal IL-6 secretion promotes the hyperplasia of mesangial cells and matrix and, thus, affects the permeability of the glomerular filtration membrane. Therefore, the detection of urinary IL-6 levels in patients with renal hematuria is beneficial for disease evaluation. A total of 82 patients with primary renal hematuria were divided into group 1 (UPr/24 h < 150 mg; pure hematuria group), group 2 (150 mg ≤ UPr/24 h ≤ 1,000 mg) and group 3 (UPr/24 h > 1,000 mg). A total of 30 normal individuals were selected as the controls. The urinary IL-6 levels were detected by the enzyme-linked immunosorbent assay (ELISA) method and a renal biopsy was conducted. The urinary IL-6 levels and renal pathological damage scores in groups 1 and 2 were significantly reduced compared with those in group 3, (P<0.001 and 0.01, respectively), with no significant difference between groups 1 and 2 (P>0.05). The correlation coefficient (r) of urinary IL-6 with 24 h urinary protein (UPr/24 h) in groups 1, 2 and 3 was 0.017, 0.045 and 0.747, respectively, and that of urinary IL-6 with renal pathological damage score was 0.627, 0.199 and 0.119, respectively. The UPr/24 h was significantly correlated with IL-6 level (r=0.7320, P<0.000). In group 1, the urinary IL-6 levels were correlated with the degree of renal pathological damage. A positive correlation was observed between urinary IL-6 levels and UPr/24 h.

## Introduction

Interleukin-6 (IL-6) is an important cytokine *in vivo*, with a wide range of biological activities. IL-6 is excreted by mononuclear cells, activated T lymphocytes and a variety of active cells. As an inflammatory mediator, IL-6 is involved in the immune injury process of glomerular diseases and is closely correlated with the incidence and development of kidney disease (1–3). *In vivo*, IL-6 has a number of sources: i) the excretion of IL-6 is increased in peripheral tissues, including muscle and fat, when the body is in an inflammatory state, and this is an important source in the circulation; ii) IL-6 is secreted by various cells, including activated macrophages, lymphocytes, fat cells, immune cells, fibroblasts and mononuclear cells, which secrete IL-6 increasingly when stimulated by tumor necrosis factor-α (TNF-α), IL-1β, bacterial endotoxins and oxidative stress (4–7). iii) IL-6 is also generated via the autocrine and paracrine systems.

Urinary IL-6 originates mainly from the kidney mesangial cells and renal tubular epithelial cells; the level of IL-6 in the urine may reflect the expression level of IL-6 in kidney tissue (8). A previous study confirmed that the sustained activation of local kidney renal artery stenosis (RAS) is one of the pathophysiological characteristics of renal artery stenosis (9). While angiotensin II may stimulate IL-6 gene expression and release of IL-6 protein by the kidneys, the latter involved in the formation of pathological changes of tubular atrophy, renal interstitial cell infiltration and mesangial cell proliferation and caused glomerulosclerosis and permeability increase of the glomerulus (5). Therefore, there may be certain correlations between the IL-6 level of renal hematuria, the severity of pathological glomerular lesions and proteinuria.

Renal hematuria (glomerular hematuria) is hematuria caused by glomerular disease (particularly glomerulonephritis). Hematuria is the main presentation of a variety of chronic kidney diseases, and is also a core feature of mesangial proliferative glomerulonephritis, IgA nephropathy and other diseases, appearing as gross hematuria, microscopic hematuria or hematuria with proteinuria. Hematuria is the early manifestation of chronic kidney disease and is often easily overlooked due to a lack of clear symptoms. Pure hematuria is common in clinical practice, which is persistent and difficult to diagnose. Renal aspiration biopsy is used to exactly diagnose isolated hematuria to guide its diagnosis and treatment. The identification of a clinical indicator, which is able to reflect the degree of severity and changes associated with isolated hematuria, is vital. Therefore, in the current study, patients with renal hematuria were selected for the observation of urinary IL-6 levels and further investigation of the correlation between urinary IL-6 levels and hematuria disease severity and changes.

## Materials and methods

### Subjects

A total of 82 patients with primary renal hematuria were selected between September 2011 and April 2012 in the Center of Urology, Beidaihe Sanatorium of Beijing Military Region (Qinhuangdao, China), including: 45 males and 37 females, average age 36±9 years. In this study, a prospective common case-control method was adopted. The renal hematuria cases were grouped according to the 24 h urinary protein (UPr/24 h) level. This study was conducted in accordance with the declaration of Helsinki and with approval from the Ethics Committee of the Center of Nephrosis of Beidaihe Sanatorium, Hebei, China. Written informed consent was obtained from all participants. According to UPr/24 h prospective case-control study method, these patients were divided into group 1 (UPr/24 h < 150 mg; pure hematuria group), group 2 (150 mg ≤ UPr/24 h ≤ 1,000 mg) and group 3 (UPr/24 h > 1,000 mg. Exclusion criteria were as follows: severe hepatic insufficiency, allergies, unexplained fever, infection, inflammatory history caused by trauma and surgery. The control group contained 30 patients, 18 males and 12 females, with an average age of 33±6 years. General information of the subjects is shown in Table I.

### Detection of urinary IL-6

Fresh morning midstream urine (2 ml) was collected before renal biopsy. After centrifuging to remove particles and polymers, an enzyme-linked immunosorbent assay (ELISA) was used to detect IL-6. The test agent was from Shanghai BlueGene Biotech Co., Ltd. (Shanghai, China; lot number J216.006.096). UPr/24 h measurement by the biuret method was completed prior to renal biopsy. Quantitation was performed prior to the application of the Olympus AU640 Chemistry Analyzer instrument (Olympus Optical Co., Ltd., Shizuoka, Japan). If the result was >3+, the solution was diluted 5-fold prior to the application of the instrument.

### Renal biopsy

A puncture needle was positioned with the aid of B-mode ultrasound imaging for renal biopsy. Paraffin sections were constructed and observation was performed by light microscopy following hematoxylin and eosin (H&E) staining, Masson’s staining, periodic acid methenamine silver (PASM) staining and periodic acid Schiff (PAS) staining, respectively. Diagnostic criteria. Clinical diagnostic criteria were as follows: urinary red blood cell count >10,000 cells/ml and morphology of red blood cells conformed to glomerular hematuria. The following diseases were excluded: urinary tract infection, prostate hypertrophy and inflammation, renal ptosis, urinary stones, tumors, renal trauma, renal tuberculosis, annexitis women, pelvic inflammatory diseases and other secondary diseases.

Standards for renal pathological damage scoring (10–12); glomerular score: i) Mesangial cell proliferation: defined as none, mild, moderate and severe proliferation according to scores of 0, 1, 2 and 3 points, respectively. ii) Matrix widening: normal status was recorded as 0 points; mild change with no significant effect on capillary loops as 1 point; moderate change with diffusing widening, and capillary loop stenosis <50% was recorded as 2 points; and severe change with diffusing widening and capillary loop stenosis >50% was recorded as 3 points. iii) Hardening changes: normal status was 0 points; focal segment distributed with glomerulosclerosis <30% was recorded as 1 point; glomerulosclerosis 30–60% was recorded as 2 points; and glomerulosclerosis >60% was recorded as 3 points. iv) Crescent formation: normal status was 0 points; focal segmental distribution <25% was 1 point; 30%–50% diffusing segmental distribution was 2 points; and 50% diffusing focal distribution was 3 points. v) Basement membrane thickening. vi) Balloon adhesion. Criteria for the evaluation of v) and vi): lesions <30% was 1 point, 30–60% was 2 points and >60% was 3 points.

### Statistical methods

Data are expressed as mean ± standard deviation; Student’s t-test analysis was used for related indicator differences among groups; linear correlation analysis was used for urinary IL-6, UPr/24 h and glomerular pathology score. SPSS 11.0 software (SPSS, Inc., Chicago, IL, USA) was used for statistical analysis. P<0.05 was considered to indicate a statistically significant result.

## Results

### Glomerular scores and urinary IL-6

The urinary IL-6 levels and renal pathological damage scores in all groups are shown in Table II. Compared with group 3, the urinary IL-6 level and renal pathological damage scores in groups 1 and 2 were significantly reduced (P<0.001 and 0.01, respectively), with no significant difference between groups 1 and 2 (P>0.05).

### Correlation analysis

**Figure 1.**
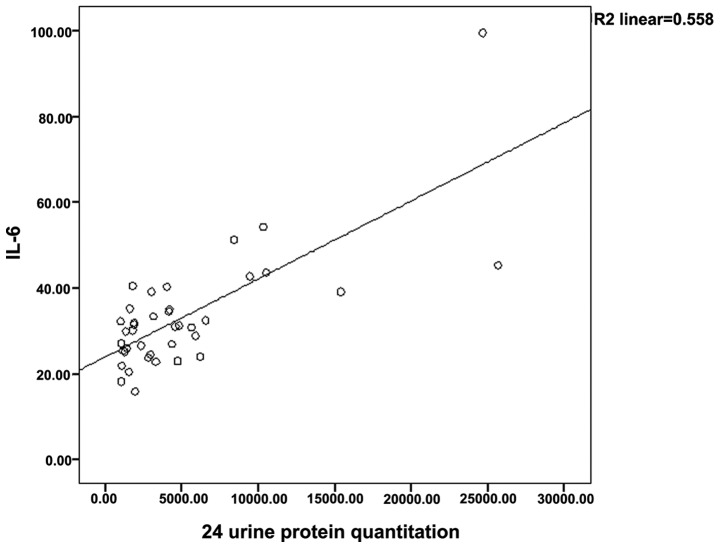
Correlation of 24-hour urinary protein quantity with the IL-6 level in group 3 (proteinuria >1,000 mg/24 h). IL-6, interleukin-6.

**Figure 2.**
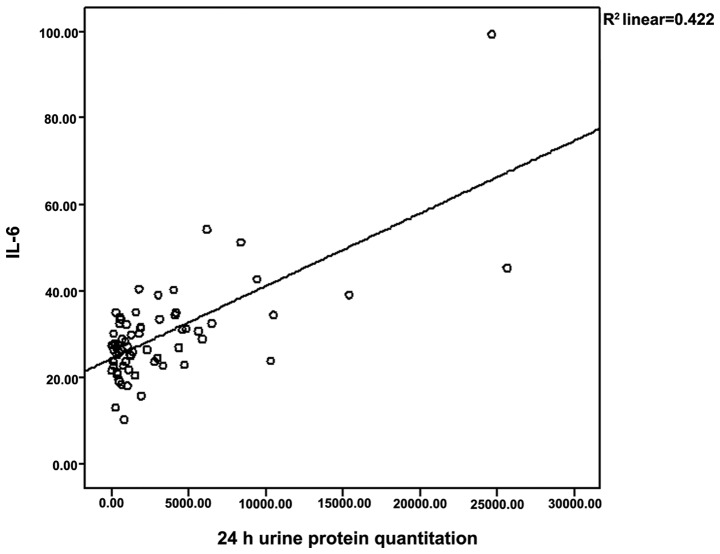
Correlation of 24-hour urinary protein quantity with the IL-6 level of 82 cases of renal hematuria. IL-6, interleukin-6.

**Figure 3.**
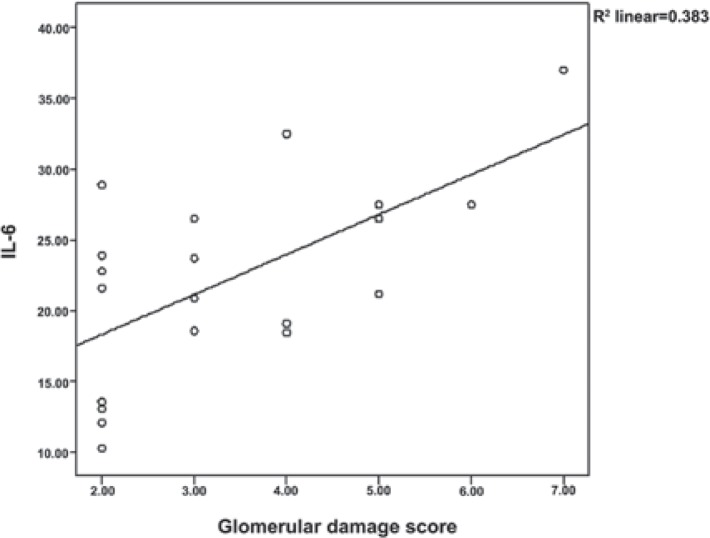
Relationship between IL-6 level and glomerular damage score in the pure hematuria group (group 1). IL-6, interleukin-6.

The correlations of urinary IL-6 levels with glomerular pathology scores and UPr/24 h are shown in Table III. Group 3, the correlation coefficients of r were 0.747 and 0.119 (between urinary IL-6 and UPr/24 h, and urinary IL-6 and pathological score of glomerular damage, respectively). Group 2, the correlation coefficients of r were 0.045 and 0.199. Group 1, the correlation coefficients of r were 0.017 and 0.627. The correlation of UPr/24 h with IL-6 in group 3 is shown in Fig. 1; the correlation coefficient r=0.747 and P<0.000. The correlation of UPr/24 h with IL-6 of all 82 cases of renal hematuria is shown in Fig. 2; the correlation coefficient r=0.732 and P<0.000. The correlation of IL-6 and glomerular damage score in the pure hematuria group (group 1) is shown in Fig. 3; the correlation coefficient r=0.627 and P<0.003, while P>0.05 for all the other groups.

## Discussion

Through the observation of urinary IL-6 levels in the three groups, the function of urinary IL-6 in renal hematuria was investigated. The results showed that the level of urinary IL-6 was positively correlated with the glomerular damage score in the pure renal hematuria group, indicating that urinary IL-6 levels correlated with glomerular pathological damage in this group. As the pathological severity was elevated, the urinary IL-6 levels also increased. This suggests that urinary IL-6 reflects the severity and changes of pure renal hematuria, providing a reference for the clinical diagnosis of asymptomatic hematuria.

A continuous increase of proteinuria is a sign of kidney damage and UPr/24 h is the gold standard for its diagnosis. In the current study, we compared the pathological score of glomerular damage in group 3 with those in groups 2 and 1, which were significantly different (P<0.01 and P<0.001, respectively). The results indicated that the increase in persistent proteinuria correlated with the aggravation of glomerular pathological damage. The r-values and P-values for the correlation of urinary IL-6 and UPr/24 h in groups 1, 2 and 3 were 0.747 and 0.000; 0.045 and 0.840; and 0.017 and 0.944, respectively. In the overall subjects with renal hematuria, UPr/24 h and IL-6 were observed to correlate (r=0.732 and P<0.000). There was no correlation between urinary IL-6 and UPr/24 h in group 2; however, urinary IL-6 was highly correlated with UPr/24 h in group 3. Previous studies (13–16) demonstrated that an angiotensin receptor blocker (ARB) was able to reduce the production of angiotensin II (Ang II) by blocking the expression of the ATI receptor in the kidney, or inhibiting the action of Ang II at the receptor level, thereby inhibiting the Ang II-mediated stimulation of superoxide formation and increase of proinflammatory cytokine release, which has a renoprotective effect independent from pressure release. Following treatment, the urinary albumin excretion rate and urinary protein and IL-6 levels were lower than those prior to treatment. Certain scholars reported that IL-6 was correlated with proteinuria in hypertensive nephropathy, suggesting that urinary protein and IL-6 levels were associated (17). In the current study, urinary IL-6 levels were positively correlated with protein quantity. IL-6 expression in the glomeruli is closely correlated with the degree of cell proliferation in the glomeruli and may regulate the mitosis of glomerular mesangial cells, promoting the proliferation of stromal hyperplasia, glomerulosclerosis and the production and release of prostaglandins, causing glomerular microvascular alterations and the increasing glomerular filtration rate (2,3,18,19). Schwartz *et al* (17) suggested that urinary IL-6 was involved in the development of pathological changes, including tubular atrophy, renal interstitial cell infiltration and mesangial cell proliferation. IL-6 may inhibit renal mononuclear cell recruitment and the proliferation of mesangial cells (20), thereby reducing atherosclerosis and improving renal tubular ischemia, reducing proteinuria and improving renal function (21). Schwartz Ihm (8) confirmed that urinary IL-6 excretion is not only correlated with the degree of glomerular inflammation reaction, but also reflects tubulointerstitial damage. The correlation of proteinuria level and IL-6 may be due to the following: in pathological cases, TNF-α, bacterial endotoxins and oxidative stress stimulate the generation of a variety of immune cells, including lymphocytes, macrophages and fibroblast cells, resulting in increased levels of IL-6 produced by immune cells (including lymphocytes, macrophages and fibroblasts), stimulated by TNF-α, bacterial endotoxin and oxidative stress, or normal renal tissue (8,22), which causes abnormal function, organizational structure and permeability increase of glomerulus, and subsequently proteinuria.

Urinary IL-6 had no correlation with glomerular damage score in the hematuria associated with the proteinuria > 1,000 mg/24 h. Urine protein is toxic to mesangial cells, so high urinary protein filtration rate would aggravate kidney damage and speed up the progress of chronic kidney disease. By contrast, glomerular score is not perfect and does not accurately reflect the causes of the extent of damage of the glomeruli. In summary, urinary IL-6 and glomerular damage score were positively correlated in the pure renal hematuria group, suggesting that urinary IL-6 may reflect the severity degree and changes of pure renal hematuria, providing a diagnostic reference for the identification of asymptomatic hematuria. In addition, urinary IL-6 levels showed a high positive correlation with protein quantity in the renal hematuria with proteinuria >1,000 mg/24 h group and the overall observation group, indicating that IL-6 may be involved in urinary protein formation.

Further studies are required to investigate whether urinary IL-6 is involved in the formation of urinary protein and to determine specific mechanisms. In addition, a continuous increase in proteinuria is a sign of kidney damage and UPr/24 h is the gold standard for diagnosis. Due to inconvenient processing and influencing factors, the real levels of urine samples would be affected. Urinary IL-6 levels showed a high positive correlation with protein quantity in the renal hematuria with proteinuria >1,000 mg/24 h group and the overall observation group, providing a reference for the diagnosis of kidney damage. However, no association was observed between urinary IL-6 level and UPr/24 h quantity in the renal hematuria with proteinuria <1,000 mg/24 h group, and this requires further investigation.

## Figures and Tables

**Table I.** General information of the subjects.

Variable	UPr/24 h > 1,000 mg	150 mg ≤ UPr/24 h ≤ 1,000 mg	UPr/24 h < 150 mg	Control group
Cases (n)	39	23	20	30
Male (%)	56	52	55	60
Age (years)^a^	38±9	36±6	35±7	33±6
Cysc (mg/l)^a^	1.98±0.71	1.66±0.62	1.51±0.52	0.86±0.31
ALB (g/l)^a^	30.45±9.05	41.03±6.59	41.22±5.67	40.5±5.83
GFR (ml/min/1.73 m^2^)^a^	48±10	75±13	82±16	93±18

UPr/24 h, 24 h urinary protein; Cysc, cystation C; ALB, serum albumin; GFR, glomerular filtration rate.

a mean±SD.

**Table II.** Glomerular pathology scores and urinary IL-6 levels.

Group	Cases (n)	Glomerular scores	Urinary IL-6 (pg/ml)
Group 1	20	3.40±1.54^b^	22.29±6.91^a^^d^
Group 2	23	3.83±2.06^c^	24.73±6.28^a^^c^
Group 3	39	5.36±2.28	33.23±13.90^a^
Control	30	-	8.50±3.70

a P<0.001 vs. control group;

b P<0.001 vs. group 3;

c P<0.01 vs. group 2;

d P>0.05 vs. group 2. IL-6, interleukin-6.

**Table III.** Correlation of urinary IL-6 with glomerular scores and UPr/24 h.

Correlation	UPr/24 h > 1000 mg		150 mg ≤ UPr/24 h ≤ 1000 mg		UPr/24 h < 150 mg	
		
	UPr/24 h	Glomerular score	UPr/24h	Glomerular score	UPr/24 h	Glomerular score
IL-6						
r	0.747	0.119	0.045	0.199	0.017	0.627
P-value	0.000	0.472	0.840	0.362	0.944	0.003

UPr/24 h, 24 h urinary protein; IL-6, interleukin-6.
